# Infectious Diseases and Maternal Morbidity and Mortality^1^

**DOI:** 10.3201/eid1011.040624_05

**Published:** 2004-11

**Authors:** Loretta P. Finnegan, Jeanne Sheffield, Harshad Sanghvi, Martha Anker

**Affiliations:** * National Institutes of Health, Bethesda, Maryland, USA;; † University of Texas Southwestern Medical Center, Dallas, Texas, USA;; ‡ JHPIEGO, Baltimore, Maryland, USA;; § World Health Organization, Geneva, Switzerland

**Keywords:** antepartum morbidity, mortality, pregnancy, pneumonia, tuberculosis, postpartum, Ebola hemorrhagic fever, SARS

## Antepartum Infectious Diseases and Maternal Morbidity and Mortality

Globally, 210 million women become pregnant each year, and 130 million births occur. However, 1,600 women die every day worldwide from childbirth; 99% of these deaths occur in the developing world. Annually, 10 million women experience related illness.

Antenatal care varies worldwide. Hemorrhage, infection, abortion, hypertension, and obstructed labor contribute to maternal illness and death. Many infectious diseases complicate antepartum care, including malaria, HIV infection, pneumonia, tuberculosis, and herpesvirus infection. Pregnancy appears to increase the severity of malaria. Recent strategies to control malaria in pregnancy include preventive treatment with antimicrobial drugs, insecticide-treated nets, and rapid diagnosis and treatment.

Throughout the world, 42 million adults and children are living with HIV/AIDS, with the largest number in sub-Saharan Africa (29.4 million). Approximately 27% of these cases are in women and adolescent girls who have been exposed through heterosexual transmission, injection drug use, sex with an injection drug user, or other unspecified risk. Eighty percent of perinatal transmission is from intrapartum transmission.

Pneumonia of any cause is less well tolerated in pregnancy. Ventilatory capacity is decreased, and the fetus may suffer hypoxemia and acidosis. Factors that can cause or lead to pneumonia in pregnancy include common viral upper respiratory infection, severe acute respiratory syndrome (SARS)–associated coronavirus, mycoplasma, chlamydia, *Haemophilus influenzae*, group A streptococcus, malaria, tuberculosis, influenza, respiratory syncytial virus, and varicella-zoster virus. Maternal or neonatal death may occur as well as intrauterine growth retardation.

Tuberculosis in pregnancy doubles the risk for prematurity and low birth weight. The risk for perinatal death increases threefold, and the risk of maternal illness increases fourfold. Miscarriage and intrapartum complications increase markedly. Antenatal care in early pregnancy permits diagnosis and treatment of many of the diseases discussed above and the management of their complications.

## Postpartum Infections in Low-Resource Settings

A number of risk factors exist for postpartum infections, including prolonged and obstructed labor, frequent vaginal examinations, prelabor rupture of the membranes, preterm birth, cesarean section, episiotomies, vacuum extractions, forceps delivery, maternal anemia, micronutrient deficiencies, poor maternal hygiene, and associated sexually transmitted diseases. Maternal illness from postpartum infections includes pelvic inflammatory disease, chronic pelvic pain, dysmenorrhea, menorrhagia, and infertility. Prevention strategies for postpartum infections include antenatal care, skilled delivery, and appropriate postpartum care. Additional strategies are minimum manipulation, high level disinfected or sterile gloves for examination, and avoiding unnecessary procedures.

Challenges in low-resource settings include lack of standards and guidelines, inappropriate training, inadequate supervision, and insufficient supplies. Infection prevention criteria should include adequate running water, cleanliness of all areas, appropriate sharps disposal, correct preparation and use of antiseptics and decontamination, correct cleaning and preparation of instruments, appropriate waste collection and disposal, and good hand washing practices.

## Women and Outbreaks of Infectious Diseases: Lessons from Ebola Hemorrhagic Fever and SARS

Gender dynamics affect transmission in the community and the empowerment of nursing staff to initiate infection control. Pregnant women and the maternity care system have special vulnerabilities; however, data disaggregated by sex and pregnancy status are lacking. The outbreaks of Ebola hemorrhagic fever and SARS have some aspects in common: both have gender-related transmission patterns; have been amplified in hospital settings; were halted by better infection control measures, isolation, and contact tracing; and lack data about pregnancies, despite nosocomial outbreaks in maternity clinics.

Ebola hemorrhagic fever is transmitted by contact with secretions or organs of infected people or animals with infected cadavers of people or animals who died. Patient isolation and contact tracing, barrier nursing methods, and safe burial practices have been used successfully to halt some outbreaks. The largest outbreaks have been amplified in healthcare settings. Most healthcare workers affected have been nurses.

The risk for Ebola hemorrhagic fever transmission is high during pregnancy. Ebola hemorrhagic fever causes spontaneous abortions with heavy bleeding in the first and second trimesters. Transmission in maternal healthcare settings was important in two of the three largest outbreaks in Yambuku and Kikwit. Recommendations for pregnant women when an Ebola hemorrhagic fever outbreak occurs include the following: surveillance of maternity clinics; careful screening of maternity patients to isolate those with complications suggestive of Ebola hemorrhagic fever; empowerment of maternity clinics staff and traditional midwives to protect themselves; and a policy on whether pregnant workers should work on Ebola hemorrhagic fever isolation wards.

Overall, 53% of SARS patients were women. The death rate is higher in men than in women. More than 21% of patients were healthcare workers, which contributed to the increased number of infected women. Little information exists on SARS and pregnancy. The World Health Organization has created an informal network of clinicians to pool experience and knowledge, develop a clinical database, and share infection control experiences.

